# Development and temporal validation of a clinical nomogram to predict delayed discharge after bariatric surgery

**DOI:** 10.1007/s00464-026-12663-2

**Published:** 2026-03-06

**Authors:** Michael T. Olson, Yun Beom Lee, Pamela Masella, Brian D. Layton

**Affiliations:** 1 Department of Bariatric Surgery, Brooke Army Medical Center, Fort Sam Houston, TX USA; 2 Department of Bariatric Surgery, Brooke Army Medical Center, 3551 Roger Brooke Drive, Fort Sam Houston, TX USA

**Keywords:** Bariatric surgery, Discharge, Enhanced recovery after surgery, Length of stay, Sleeve gastrectomy, Roux-en-Y gastric bypass

## Abstract

**Background:**

Enhanced recovery after surgery (ERAS) pathways have reduced hospital stays after bariatric surgery, yet a subset of patients still require prolonged hospitalization. We aimed to identify predictors of delayed discharge and develop a validated clinical nomogram to estimate the likelihood of > 1 day postoperative stay.

**Methods:**

We performed a retrospective cohort study of consecutive adults undergoing primary or revisional minimally invasive sleeve gastrectomy or Roux-en-Y gastric bypass at a single military treatment facility from 01/01/2022 to 12/31/2024. Demographic, comorbidity, operative, and perioperative data were abstracted. The 2023 cohort served as the development set for univariable and multivariable logistic regression to identify predictors of delayed discharge (> 1 day). A clinical nomogram was constructed from independent predictors and internally validated via bootstrap resampling (200 iterations). Temporal validation was performed on 2022 and 2024 cohorts.

**Results:**

Among 281 patients (mean age 47.2 ± 11.3 years; mean BMI 40.5 ± 6.0 kg/m^2^; 26.7% male), 141 (50.2%) experienced delayed discharge. Independent predictors included operative time > 150 min (OR 3.00, 95% CI 1.14–8.09), overnight hydromorphone use (OR 3.78, 95% CI 1.40–11.0), ≥ 1 overnight antiemetic dose (OR 2.55, 95% CI 1.04–6.27), postoperative day (POD) 0 oral intake < 200 mL (OR 2.43, 95% CI 1.01–6.01), and POD 1 hemoglobin decrease ≥ 2 g/dL (OR 4.16, 95% CI 1.25–15.3). The final five-variable model demonstrated strong discrimination (AUC 0.77; bias-corrected C-index 0.74) and calibration (Hosmer–Lemeshow *p* = 0.17). Temporal validation confirmed robust performance (AUC 0.77–0.87). In sensitivity analysis, model discrimination remained high for both primary (AUC 0.79) and revisional cases (AUC 0.88). A web-based Shiny risk calculator was developed for bedside use (https://michaeltolson.shinyapps.io/bariatric-delayed-discharge-2023/).

**Conclusions:**

A five-variable nomogram accurately predicts delayed discharge following bariatric surgery and demonstrated strong temporal validation. This tool may aid individualized discharge planning.

**Supplementary Information:**

The online version contains supplementary material available at 10.1007/s00464-026-12663-2.

Bariatric surgery remains the most effective long-term therapy for obesity and its metabolic complications [1]. With the adoption of Enhanced Recovery After Surgery (ERAS) pathways, same-day and next-day discharge have become achievable and safe for many patients undergoing minimally invasive sleeve gastrectomy (SG) and Roux-en-Y gastric bypass (RYGB) [2–4]. Despite these advances, a proportion of patients still require prolonged hospitalization, commonly for persistent nausea, inadequate pain control, or insufficient oral intake.

Hospital length of stay (LOS) after bariatric surgery reflects both clinical recovery and institutional efficiency, serving as a process-quality metric in ERAS programs. Prolonged LOS increases resource utilization [5] and is associated with an increase in early readmissions [6, 7]. While prior studies have identified individual risk factors for longer stays, such as select comorbidities [8, 9], lower postoperative oral intake [10], and postoperative complications [11], no validated model currently integrates these variables to predict delayed discharge at the patient level.

The present study aimed to (1) identify predictors of delayed discharge following bariatric surgery, (2) develop a clinical nomogram for individualized risk estimation, and (3) validate its performance temporally at our institution. We hypothesized that select preoperative or perioperative factors would predict the likelihood of > 1 day hospital stays.

## Materials and methods

### Study design and setting

This was a retrospective, single-center cohort study conducted at a United States military treatment facility. Institutional review board approval was obtained prior to data collection.

### Patient selection

The study included all adults (≥ 18 years) who underwent bariatric surgery between January 1, 2022 and December 31, 2024, including both primary and revisional (or conversion) minimally invasive SG and RYGB. All procedures were performed by two fellowship-trained bariatric surgeons. Exclusion criteria included other bariatric-related cases performed by both surgeons in the same time period, including isolated adjustable gastric band removals, bariatric reversal procedures, remnant gastrectomy, or procedures performed for bariatric complications, such as fistula takedown.

### Institutional ERAS protocol

All patients were managed according to the same standardized ERAS protocol for bariatric procedures. Preoperatively, patients received multimodal analgesia consisting of intravenous acetaminophen and gabapentin, as well as weight-based enoxaparin for venous thromboembolism (VTE) chemoprophylaxis. General anesthesia was used in all cases, and all patients received intraoperative transversus abdominis plane block, performed by the operating surgeon, with combination 0.25% bupivacaine (Marcaine) and liposomal bupivacaine (Exparel) for additional pain control.

Postoperatively on the ward, patients remained nil per os for six hours before starting a stage 1 bariatric diet on postoperative day (POD) 0, consisting of clear liquids in small, frequent volumes. Early ambulation was emphasized, with patients encouraged to ambulate at least once on POD 0. All patients received maintenance intravenous fluids at a standard rate of 125 mL/hour until clinical assessment during morning rounds on POD 1. If patients were symptomatic for oral intake intolerance, maintenance intravenous fluids were continued until reassessment at end-of-day POD 1. Standardized, multimodal, opioid-sparing analgesia was used postoperatively and supplemented by as-needed antiemetics. Antiemetics were not scheduled routinely; rather, administration was symptom-driven. Patients were instructed to track oral intake and maintain at least 1 fluid ounce per hour overnight. On the morning of POD 1, a complete blood count (CBC) was obtained. If the hemoglobin had decreased by ≥ 2 g/dL from baseline, a repeat CBC was obtained at noon to evaluate for ongoing decline or occult bleeding. If hemoglobin remained stable from baseline, weight-based enoxaparin was initiated for VTE chemoprophylaxis. Routine upper gastrointestinal (GI) contrast studies were not performed, except when clinically indicated (eg, suspicion for leak or obstruction).

Patients were discharged once they tolerated oral intake, which meant drinking 4–5 fluid ounces per hour, had adequate pain control on oral medications, and met ambulation and hemodynamic stability criteria. As a practice, our institution does not routinely discharge patients same-day from bariatric surgery.

### Variables collected and primary outcome

Demographics, preoperative clinical data, operative indices, and perioperative variables were abstracted from the electronic health record. Key demographic and preoperative variables included age, sex, race, home of record distance from hospital (miles), marital status, body mass index (BMI), comorbidities (e.g., obstructive sleep apnea [OSA], hypertension, diabetes mellitus, hyperlipidemia [HLD], gastroesophageal reflux disease [GERD], chronic pain, psychiatric diagnoses, among others), and prior bariatric surgery. Operative variables included procedure type, surgical approach, concurrent hiatal hernia repair, operative time, and intraoperative complications. Perioperative variables included oral intake on POD 0 through POD 1, oral and intravenous opioid administration (and quantified with morphine equivalents), antiemetic doses, hemoglobin change, and length of stay. ‘Overnight’ was defined as time from 1900 to 0700 on POD 0 to 1, which corresponded to nursing shifts and facilitated the most accurate intake/output assessment. The primary outcome was delayed discharge, defined as hospital stay > 1 day following the index procedure.

### Cohort definitions

Patients who underwent bariatric surgery between January 1, 2023 and December 31, 2023 were included the ‘developmental’ cohort, which was used for the univariable analysis, multivariable analysis, model derivation, and internal validation. These patients represented the largest cohort in terms of annual case volume. Two separate cohorts were then derived from patients who underwent bariatric surgery between January 1, 2022 and December 31, 2022, and January 1, 2024 and December 31, 2024, used for temporal validation.

### Statistical analysis

Continuous variables were summarized as mean ± SD or median [IQR] and compared using t-tests or Wilcoxon rank-sum tests, as appropriate. Categorical variables were compared using *χ*^2^ or Fisher’s exact tests. For multi-year comparisons across the 2022, 2023, and 2024 cohorts, continuous variables were summarized as mean ± SD or median [IQR] and compared using one-way ANOVA or Kruskal–Wallis tests, depending on normality.

Univariable logistic regression was first performed within the developmental cohort, as mentioned above, to identify variables associated with delayed discharge, using a liberal inclusion threshold of *p* < 0.10. Significant predictors and clinically relevant covariates were subsequently entered into a multivariable logistic regression model. Independent binary predictors from the final model were incorporated into a parsimonious nomogram reflecting relative variable weights. Odds ratios (OR) with 95% confidence intervals (CI) were reported.

Model performance was evaluated using receiver operating characteristic (ROC) analysis and corresponding area under the curve (AUC, equivalent to the concordance index [C-index]) to quantify discrimination. Optimal probability cutoffs were determined using the Youden index to balance sensitivity and specificity. Temporal validation was performed in the 2022 and 2024 cohorts to assess out-of-sample discrimination, calibration, and classification accuracy (sensitivity, specificity, positive predictive value [PPV], and negative predictive value [NPV]). A calibration curve was generated for each cohort by plotting observed vs. predicted event rates across deciles of predicted probability, and overall model fit was assessed using the Hosmer–Lemeshow goodness-of-fit test. A non-significant result (*p* > 0.05) was interpreted as evidence of good calibration.

A prespecified sensitivity analysis was conducted to evaluate model robustness by procedure type (primary vs. revisional and conversion operations). Additionally, a pooled analysis across all study years was performed to determine overall discrimination and calibration performance.

All statistical analyses were conducted in R (version 4.3.3) (R Foundation for Statistical Computing, Vienna, Austria). Visualization of ROC and calibration curves was performed using ggplot2. A web-based, interactive risk calculator was developed using the *shiny* package to allow bedside estimation of delayed discharge probability.

### Reporting standards

This study was designed, conducted, and reported in accordance with the Strengthening the Reporting of Observational Studies in Epidemiology (STROBE) guidelines for observational research [12].

## Results

### Overall cohort characteristics

A total of 293 patients underwent bariatric surgery at our institution during the study period. Twelve patients were excluded based on procedure type: adjustable gastric band removal (*N* = 7), bariatric reversal procedure (*N* = 2), remnant gastrectomy (*N* = 1), and gastrogastric fistula repair (*N* = 2). A total of 281 patients were included in the study. Overall, the mean age was 47.2 ± 11.3 years, and 26.7% were male. Most patients were married (82.9%) and white race (51.2%). The mean preoperative BMI was 40.5 ± 6.0 kg/m^2^, and the most common comorbidities were GERD (68.0%), OSA (66.9%), and HLD (61.2%). Most cases were approached laparoscopically (98.2%). There were 157 (55.9%) RYGB and 124 (44.1%) SG performed. The mean LOS was 1.6 ± 0.9 days. The overall rate of delayed discharge was 50.2%. Table 1 summarizes the demographics and preoperative characteristics for the entire cohort; Table 2 shows their operative and perioperative outcomes.

**Table 1:** Overall cohort by demographics and preoperative characteristics

Variable	Overall (*N* = 281)	2022 (*N* = 83)	2023 (*N* = 122)	2024 (*N* = 76)	p-value
*Demographics*					
Age (years)	47.2 ± 11.3	48.6 ± 11.5	46.1 ± 10.7	47.4 ± 12.0	0.192
Male sex	75 (26.7%)	24 (28.9%)	30 (24.6%)	21 (27.6%)	0.771
Race					**0.002**
White	144 (51.2%)	48 (57.8%)	59 (48.4%)	37 (48.7%)	
Black	46 (16.4%)	1 (1.2%)	27 (22.1%)	18 (23.7%)	
Hispanic	23 (8.2%)	9 (10.8%)	10 (8.2%)	4 (5.3%)	
Other	68 (24.2%)	25 (30.1%)	26 (21.3%)	17 (22.4%)	
Married	233 (82.9%)	73 (88.0%)	101 (82.8%)	59 (77.6%)	0.225
Home distance (miles)	82.1 ± 245.8	85.2 ± 225.9	83.6 ± 247.0	76.1 ± 266.9	0.172
Military retiree status	123 (43.8%)	38 (45.8%)	56 (45.9%)	29 (38.2%)	0.513
*Preoperative characteristics*					
Preoperative weight (kg)	112.6 ± 23.5	115.4 ± 21.2	111.4 ± 24.5	111.3 ± 24.3	0.298
Preoperative height (cm)	165.9 ± 10.0	167.8 ± 10.1	164.3 ± 10.3	166.3 ± 9.2	0.146
Preoperative BMI (kg/m^2^)	40.5 ± 6.0	40.7 ± 5.2	40.6 ± 6.2	40.1 ± 6.5	0.857
Ideal body weight (kg)	69.1 ± 8.3	70.7 ± 8.5	67.8 ± 8.3	69.3 ± 7.8	0.146
Excess body weight (kg)	43.6 ± 20.9	44.7 ± 16.2	43.6 ± 24.2	42.1 ± 19.7	0.723
ASA class	3 (2–3)	3 (2–3)	3 (2–3)	3 (2–3)	1.000
Prediabetes	99 (35.2%)	20 (24.1%)	45 (36.9%)	34 (44.7%)	**0.022**
Type 2 diabetes mellitus	52 (18.5%)	12 (14.5%)	20 (16.4%)	20 (26.3%)	0.114
Obstructive sleep apnea	188 (66.9%)	59 (71.1%)	83 (68.0%)	46 (60.5%)	0.346
Hypertension	138 (49.1%)	37 (44.6%)	57 (46.7%)	44 (57.9%)	0.191
Hyperlipidemia	172 (61.2%)	35 (42.2%)	86 (70.5%)	51 (67.1%)	** < 0.001**
Nonalcoholic fatty liver disease	70 (24.9%)	15 (18.1%)	32 (26.2%)	23 (30.3%)	0.187
Gastroesophageal reflux disease	191 (68.0%)	55 (66.3%)	83 (68.0%)	53 (69.7%)	0.896
Atrial fibrillation	8 (2.8%)	1 (1.2%)	4 (3.3%)	3 (3.9%)	0.542
Chronic pain	89 (31.7%)	18 (21.7%)	38 (31.1%)	33 (43.4%)	**0.013**
Psychiatric diagnoses	172 (61.2%)	35 (42.2%)	86 (70.5%)	51 (67.1%)	** < 0.001**
Number of preoperative comorbidities	4.2 ± 1.8	3.46 (1.68)	4.38 (1.71)	4.71 (1.67)	** < 0.001**
Smoking history	78 (27.8%)	12 (14.5%)	46 (37.7%)	20 (26.3%)	**0.001**
Bariatric surgical history	47 (16.7%)	11 (13.3%)	16 (13.1%)	20 (26.3%)	**0.032**
Preoperative hemoglobin A1c	5.8 ± 1.0	5.6 ± 0.7	5.8 ± 1.0	6.0 ± 1.2	**0.013**
Preoperative lipid profile					
Total cholesterol	183.8 ± 39.7	178.9 ± 39.2	189.4 ± 40.0	180.2 ± 39.1	0.103
High density lipoprotein	47.9 ± 12.7	46.0 ± 12.3	49.0 ± 12.7	48.2 ± 13.2	0.188
Low density lipoprotein	109.8 ± 34.8	106.8 ± 35.2	113.0 ± 35.2	107.8 ± 33.8	0.284
Triglycerides	138.8 ± 125.0	130.6 ± 58.2	150.3 ± 172	129.2 ± 78.9	0.467

Bolded values are statistically significant (*p* < 0.05)

Categorical variables are reported as N (%); continuous variables are reported as mean (SD) or median (IQR)

*ASA* American Society of Anesthesiologists, *BMI* body mass index

**Table 2:** Overall cohort by operative and perioperative outcomes

Variables	Overall (*N* = 281)	2022 (*N* = 83)	2023 (*N* = 122)	2024 (*N* = 76)	p-value
*Operative characteristics*					
Surgical approach					
Laparoscopic	276 (98.2%)	83 (100.0%)	117 (95.9%)	76 (100.0%)	**0.036**
Robotic-assisted	5 (1.8%)	0 (0.0%)	5 (4.1%)	0 (0.0%)	**0.036**
Procedure type					
Sleeve gastrectomy	124 (44.1%)	38 (45.8%)	54 (44.3%)	32 (42.1%)	0.896
Roux-en-Y gastric bypass	157 (55.9%)	45 (54.2%)	68 (55.7%)	44 (57.9%)	0.896
Revisional/conversion surgery	47 (16.7%)	11 (13.3%)	16 (13.1%)	20 (26.3%)	**0.032**
Concurrent hiatal hernia repair	116 (41.3%)	33 (39.8%)	50 (41.0%)	33 (43.4%)	0.893
Operative time, minutes	143.7 ± 58.7	142.9 ± 48.7	137.2 ± 52.5	155.1 ± 75.0	0.246
Intraoperative complications	10 (3.6%)	1 (1.2%)	4 (3.3%)	5 (6.6%)	0.184
*Perioperative outcomes*					
Overnight antiemetic doses	0.8 ± 1.2	0.6 ± 1.4	0.9 ± 1.2	0.8 ± 0.9	**0.013**
Overnight oxycodone use (mg)	6.6 ± 7.1	8.4 ± 9.3	5.9 ± 6.1	5.6 ± 5.2	0.468
Overnight hydromorphone use (mg)	0.2 ± 0.3	0.2 ± 0.4	0.2 ± 0.3	0.2 ± 0.3	0.804
Overnight morphine equivalents (mg)	11.2 ± 10.9	14.7 ± 14.0	10.1 ± 9.6	9.2 ± 7.8	0.146
Overnight oral intake (mL)	194.5 ± 199.0	167.7 ± 202.7	199.9 ± 196.6	215.3 ± 198.0	0.121
POD 1 total oral intake (mL)	577.1 ± 380.6	473.3 ± 317.8	645.0 ± 376.1	581.5 ± 427.9	**0.003**
Total oxycodone use (mg)	19.2 ± 19.8	26.6 ± 26.0	16.3 ± 17.3	15.9 ± 12.6	**0.037**
Total hydromorphone use (mg)	0.6 ± 0.8	0.7 ± 0.7	0.6 ± 0.9	0.7 ± 0.8	**0.037**
Total morphine equivalents (mg)	33.1 ± 32.0	46.8 ± 41.3	27.5 ± 27.8	27.3 ± 20.5	**0.002**
Hemoglobin decrease ≥ 2 g/dL on POD 1	51 (18.1%)	11 (13.3%)	24 (19.7%)	16 (21.1%)	0.375
LOS, days	1.6 ± 0.9	1.6 ± 0.8	1.6 ± 0.8	1.8 ± 1.1	0.625
Delayed discharge (> 1 day)	141 (50.2%)	37 (44.6%)	64 (52.5%)	39 (51.3%)	0.517

Bolded values are statistically significant (*p* < 0.05)

Categorical variables are reported as N (%). Continuous variables are reported as mean (SD)

*LOS* length of stay, *mg* milligram, *mL* milliliter, *POD* postoperative day

### Comparison by length of stay

Table 3 compares demographics, preoperative characteristics, operative, and perioperative outcomes by LOS. Female patients and dependents of military members (vs. retirees) were more frequent in the delayed discharge cohort. Baseline comorbidities were largely similar between groups. Procedure type, rates of concurrent hiatal hernia repair, and intraoperative complications also did not differ between groups. Patients with delayed discharge were more likely to have operative time > 150 min (*p* = 0.004) and higher perioperative antiemetic and opioid use (*p* < 0.001). They demonstrated lower mean oral intake overnight and through POD 1 (*p* < 0.001). Lastly, they were more likely to experience a ≥ 2 g/dL decrease in hemoglobin from baseline on POD 1 (*p* < 0.001).

**Table 3:** Comparison of outcomes by length of stay

Variable	LOS 1 day (*N* = 141)	LOS > 1 day (*N* = 140)	p-value
*Demographics*			
Age (years)	46.5 ± 10.8	47.90 ± 11.78	0.291
Male sex	50 (35.5%)	25 (17.9%)	**0.001**
White race	77 (54.6%)	67 (47.9%)	0.284
Married	122 (86.5%)	111 (79.3%)	0.115
Home distance (miles)	86.8 ± 249.2	77.3 ± 243.1	0.746
Military retiree status	75 (53.2%)	48 (34.3%)	**0.002**
*Preoperative characteristics*			
Preoperative BMI (kg/m^2^)	40.3 ± 5.2	40.7 ± 6.7	0.506
ASA class	3 (2–3)	3 (2–3)	1.000
Prediabetes	49 (34.8%)	50 (35.7%)	0.901
Type 2 diabetes mellitus	29 (20.6%)	23 (16.4%)	0.443
Obstructive sleep apnea	98 (69.5%)	90 (64.3%)	0.377
Hypertension	79 (56.0%)	59 (42.1%)	**0.023**
Hyperlipidemia	91 (64.5%)	81 (57.9%)	0.272
Nonalcoholic fatty liver disease	32 (22.7%)	38 (27.1%)	0.411
Gastroesophageal reflux disease	92 (65.2%)	99 (70.7%)	0.371
Atrial fibrillation	5 (3.5%)	3 (2.1%)	0.723
Chronic pain	40 (28.4%)	49 (35.0%)	0.250
Psychiatric diagnoses	82 (58.2%)	90 (64.3%)	0.328
Number of preoperative comorbidities	4.2 ± 1.8	4.2 ± 1.7	0.715
Smoking history	41 (29.1%)	37 (26.4%)	0.690
*Operative characteristics*			
Laparoscopic approach	139 (98.6%)	137 (97.9%)	0.684
Sleeve gastrectomy	67 (47.5%)	57 (40.7%)	0.280
Roux-en-Y gastric bypass	74 (52.5%)	83 (59.3%)	0.280
Revisional/conversion surgery	20 (14.2%)	27 (19.3%)	0.267
Concurrent hiatal hernia repair	57 (40.4%)	59 (42.1%)	0.809
Operative time (minutes)	133.7 ± 46.1	153.8 ± 67.8	**0.004**
Intraoperative complications	4 (2.8%)	6 (4.3%)	0.541
*Perioperative outcomes*			
Overnight antiemetic doses	0.4 ± 0.6	1.1 ± 1.5	** < 0.001**
Overnight oxycodone use (mg)	6.7 ± 6.9	6.5 ± 7.2	0.811
Overnight hydromorphone use (mg)	0.1 ± 0.2	0.3 ± 0.4	** < 0.001**
Overnight morphine equivalents (mg)	11.0 ± 10.7	11.4 ± 11.1	0.754
Overnight oral intake (mL)	275.7 ± 214.7	113.2 ± 141.6	** < 0.001**
POD 1 total oral intake (mL)	724.2 ± 364.5	430.0 ± 338.0	** < 0.001**
Total oxycodone use (mg)	13.6 ± 13.6	24.9 ± 23.2	** < 0.001**
Total hydromorphone use (mg)	0.5 ± 0.5	0.8 ± 1.0	** < 0.001**
Total morphine equivalents (mg)	23.9 ± 21.9	42.4 ± 37.5	** < 0.001**
Hemoglobin decrease ≥ 2 g/dL on POD 1	8 (5.7%)	43 (30.7%)	** < 0.001**
LOS, days	1.0 ± 0.0	2.3 ± 0.8	** < 0.001**

Bolded values are statistically significant (*p* < 0.05)

Categorical variables are reported as N (%). Continuous variables are reported as mean (SD)

*ASA* American Society of Anesthesiologists, *BMI* body mass index, *LOS* length of stay, *mg* milligram, *mL* milliliter, *POD* postoperative day

### Comparison by developmental and validation cohorts

Demographics, preoperative characteristics, operative, and perioperative outcomes were compared between annual cohorts to assess for differences between the development and temporal validation groups. White race was less frequent in the latter two years, while ‘other’ races comprised a greater proportion of patients from 2022; otherwise, demographics were comparable. Prediabetes (*p* = 0.022), smoking history (*p* = 0.001), chronic pain (*p* = 0.013), and psychiatric diagnoses (*p* < 0.001) were more frequent in 2023 and 2024 compared to 2022. Mean number of comorbidities uptrended from 2022 to 2024. More revisional/conversion cases were performed in 2024 (*p* = 0.032) than the earlier two years. Opioid use was higher in patients from 2022 compared to the latter two years. Operative time, mean LOS, and rates of delayed discharge were comparable across all groups. Tables 1 and 2 can be referenced to view differences between these three cohorts.

### Univariable logistic regression analysis

On univariable logistic regression, higher BMI and unmarried status trended toward significance. No associations were found for age, sex, race, or specific procedure type. Several perioperative factors were significantly associated with delayed discharge. Operative time, analyzed continuously, was predictive of delayed discharge (OR 1.01, 95% CI 1.00–1.02, *p* = 0.006), as was an operative duration > 150 min (OR 2.53, 95% CI 1.16–5.50, *p* = 0.022). Delayed discharge was also predicted by overnight hydromorphone use (OR 2.94, 95% CI 1.42–6.08, *p* = 0.004), receipt of ≥ 1 overnight antiemetic dose (OR 2.54, 95% CI 1.23–5.25, *p* = 0.012), POD 0 oral intake < 200 mL (OR 3.92, 95% CI 1.85–8.27, *p* < 0.001), and POD 1 hemoglobin decrease ≥ 2 g/dL (OR 3.39, 95% CI 1.30–10.0, *p* = 0.017). Table 4 shows the complete univariable logistic regression analysis.

**Table 4:** Univariable logistic regression analysis of predictors of delayed discharge

Category	Variable	OR	95% CI	p-value
Demographics	Age (years)	1.00	0.96–1.04	0.901
	Male sex	0.51	0.22–1.18	0.119
	White race	0.59	0.29–1.21	0.153
	Married	0.38	0.13–1.09	0.062
	Home distance (miles)	1.00	1.00–1.00	0.924
	Military retiree status	0.56	0.27–1.14	0.113
Preoperative	Body mass index (kg/m^2^)	1.06	1.00–1.14	0.058
	Hypertension	0.68	0.33–1.39	0.292
	Comorbidity count	1.04	0.85–1.27	0.681
Operative	Sleeve gastrectomy	0.56	0.27–1.18	0.116
	Roux-en-Y gastric bypass	1.79	0.87–3.70	0.116
	Revisional/conversion surgery	1.19	0.41–3.47	0.745
	Operative time (min)	1.01	1.00–1.02	**0.006**
	Operative time > 150 min	2.53	1.16–5.50	**0.022**
Perioperative	Overnight hydromorphone use (any)	2.94	1.42–6.08	**0.004**
	≥ 1 overnight antiemetic dose	2.54	1.23–5.25	**0.012**
	POD 0 oral intake < 200 mL	3.92	1.85–8.27	** < 0.001**
	Hemoglobin decrease ≥ 2 g/dL POD 1	3.39	1.30–10.0	**0.017**

Bolded values are statistically significant (*p* < 0.05)

*CI* confidence interval, *OR* odds ratio, *POD* postoperative day

### Multivariable logistic regression analysis and nomogram development

On multivariable logistic regression, several perioperative variables remained independent predictors of delayed discharge following bariatric surgery (Table 5). Operative time > 150 min (adjusted OR 3.00, 95% CI 1.14–8.09, *p* = 0.030), any overnight hydromorphone use (adjusted OR 3.78, 95% CI 1.40–11.0, *p* = 0.011), receipt of ≥ 1 overnight antiemetic dose (adjusted OR 2.55, 95% CI 1.04–6.27, *p* = 0.046), POD 0 oral intake < 200 mL (adjusted OR 2.43, 95% CI 1.01–6.01, *p* = 0.048), and POD 1 hemoglobin decrease ≥ 2 g/dL (adjusted OR 4.16, 95% CI 1.25–15.3, *p* = 0.026) were all significantly associated with delayed discharge. Higher preoperative BMI was independently associated with delayed discharge (adjusted OR 1.12 per kg/m^2^, 95% CI 1.03–1.24, *p* = 0.017), however, when evaluated as a continuous predictor, its discriminative capacity was poor (AUC = 0.51, 95% CI 0.44–0.58) with a Youden-optimal cutoff of 45 kg/m^2^ (sensitivity 25%, specificity 85%) (Fig. S1).

**Table 5:** Multivariable logistic regression analysis of predictors of delayed discharge

Category	Variable	Adjusted OR	95% CI	p-value
Demographic	Age (years)	1.01	0.97–1.06	0.596
	Male sex	0.37	0.12–1.08	0.067
	White race	0.62	0.25–1.54	0.286
	Married	0.58	0.17–1.92	0.362
Preoperative	Body mass index (kg/m^2^)	1.12	1.03–1.24	**0.017**
Operative	Operative time > 150 min	3.00	1.14–8.09	**0.030**
Perioperative	Overnight hydromorphone use (any)	3.78	1.40–11.0	**0.011**
	≥ 1 overnight antiemetic dose	2.55	1.04–6.27	**0.046**
	POD 0 oral intake < 200 mL	2.43	1.01–6.01	**0.048**
	Hemoglobin decrease ≥ 2 g/dL POD 1	4.16	1.25–15.3	**0.026**

Bolded values are statistically significant (*p* < 0.05)

*CI*, confidence interval, *OR* odds ratio, *POD* postoperative day

**Fig. 1:**
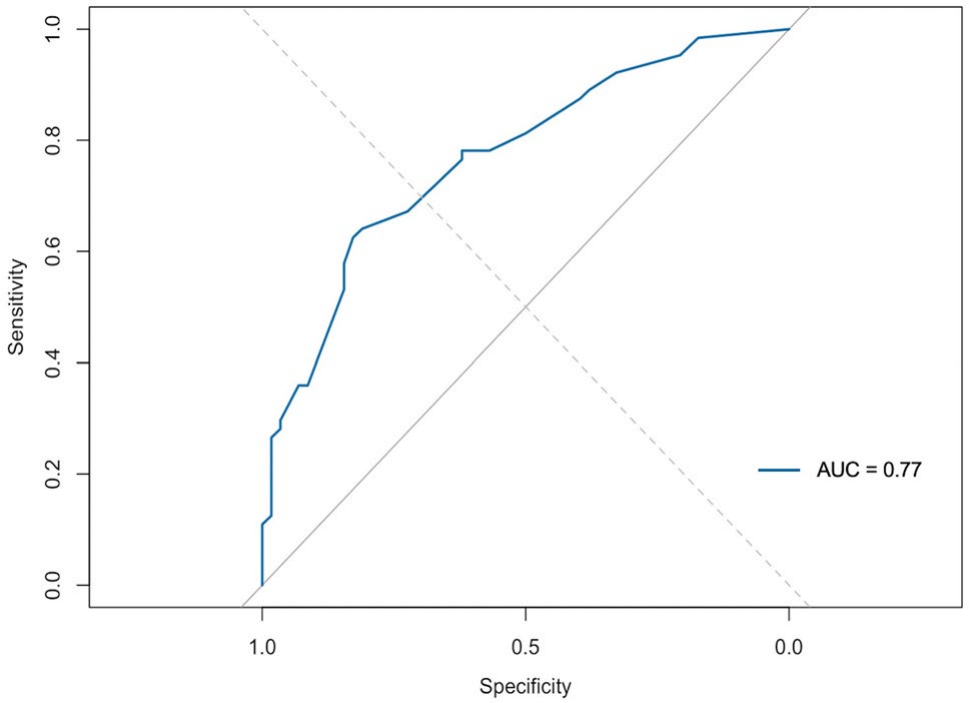
Receiver operating characteristic (ROC) curve for delayed discharge. ROC analysis of the multivariable model predicting delayed discharge after bariatric surgery. The model demonstrated good discrimination with an area under the curve (AUC) equal to 0.77, indicating reliable separation between patients with and without delayed discharge

**Fig. 2:**
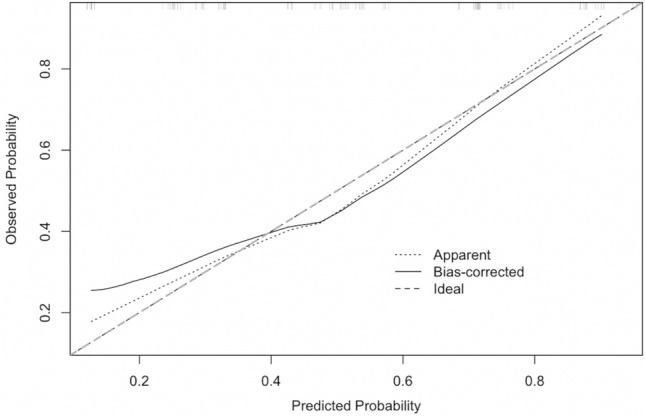
Internal calibration of the delayed discharge model. Bootstrap-corrected calibration plot comparing predicted and observed probabilities of delayed discharge. The solid line represents bias-corrected calibration, the dotted line the apparent performance, and the dashed line the ideal 45° reference. The model showed close agreement between predicted and observed risk, supporting good internal validity

**Fig. 3:**
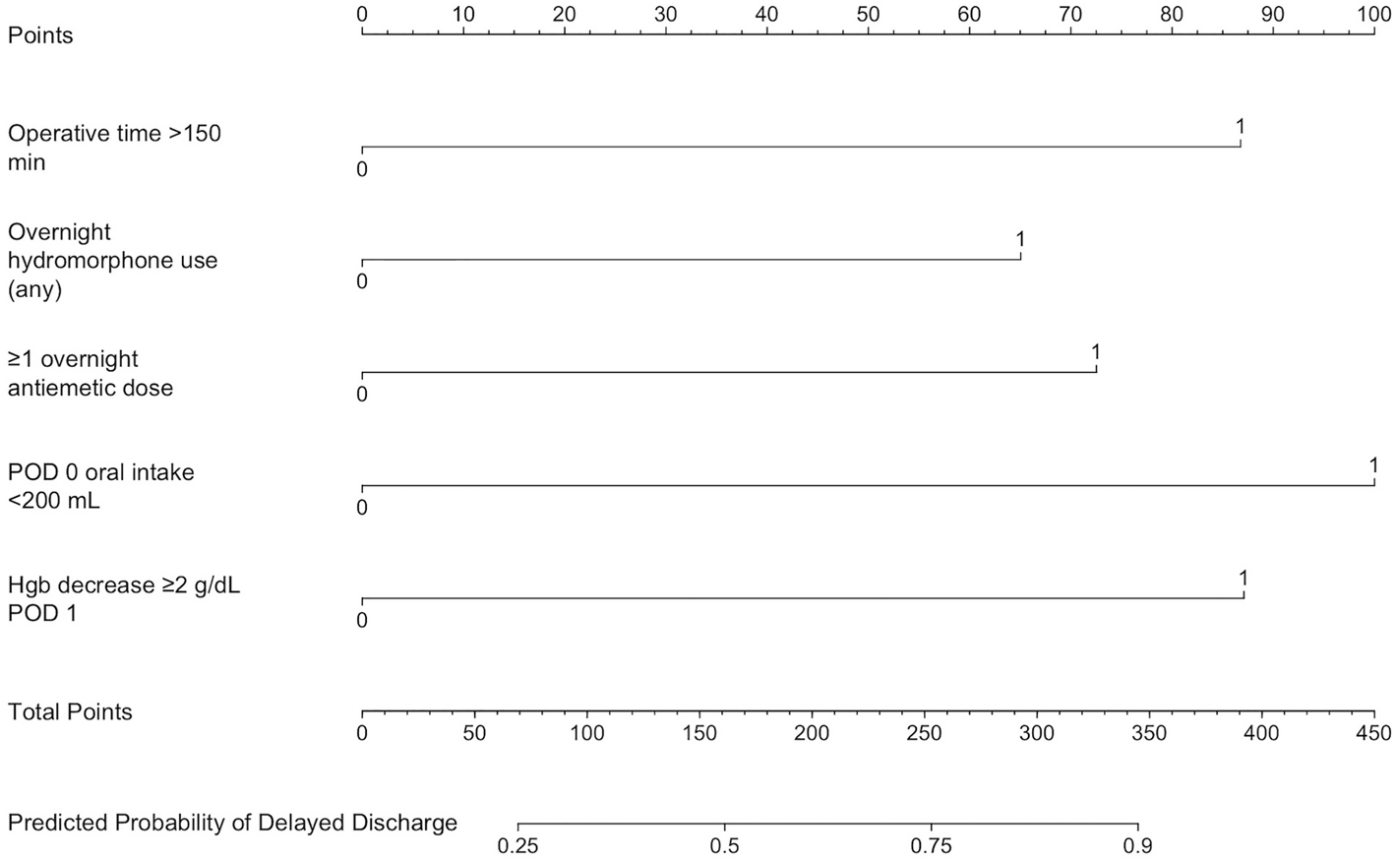
Nomogram for predicting delayed discharge. Nomogram derived from the multivariable logistic regression model to estimate the probability of delayed discharge (> 1 postoperative day). For an individual patient, each predictor variable (operative time > 150 min, overnight hydromorphone use, ≥ 1 overnight antiemetic dose, postoperative day 0 [POD0] oral intake < 200 mL, and hemoglobin decrease ≥ 2 g/dL on POD1) is assessed as present or absent. A vertical line is drawn from each positive predictor to the top “Points” scale to assign a point value. Points are then summed across all predictors and located on the “Total Points” axis. A vertical line drawn downward from the total point value corresponds to the estimated probability of delayed discharge on the bottom scale. Total point values extending beyond the displayed probability range indicate a very high likelihood (> 90%) of delayed discharge

Given its marginal discriminatory performance, BMI was excluded from the final nomogram, which prioritized binary, perioperative predictors measured within the first 24 postoperative hours. The final five-variable model–operative time > 150 min, any overnight hydromorphone use, ≥ 1 overnight antiemetic dose, POD 0 oral intake < 200 mL, and POD 1 hemoglobin decrease ≥ 2 g/dL–demonstrated good discrimination (AUC = 0.77, 95% CI 0.69–0.85) and internal validity (bias-corrected C-index = 0.74 following 200 bootstrap resamples) (Fig. 1). Calibration analysis showed close agreement between predicted and observed risk (Fig. 2). A simplified nomogram (Fig. 3) was developed to facilitate bedside application, achieving an optimal probability threshold of 0.56 with sensitivity 0.63, specificity 0.83, PPV 0.80, and NPV 0.67.

### Temporal validation

**Fig. 4:**
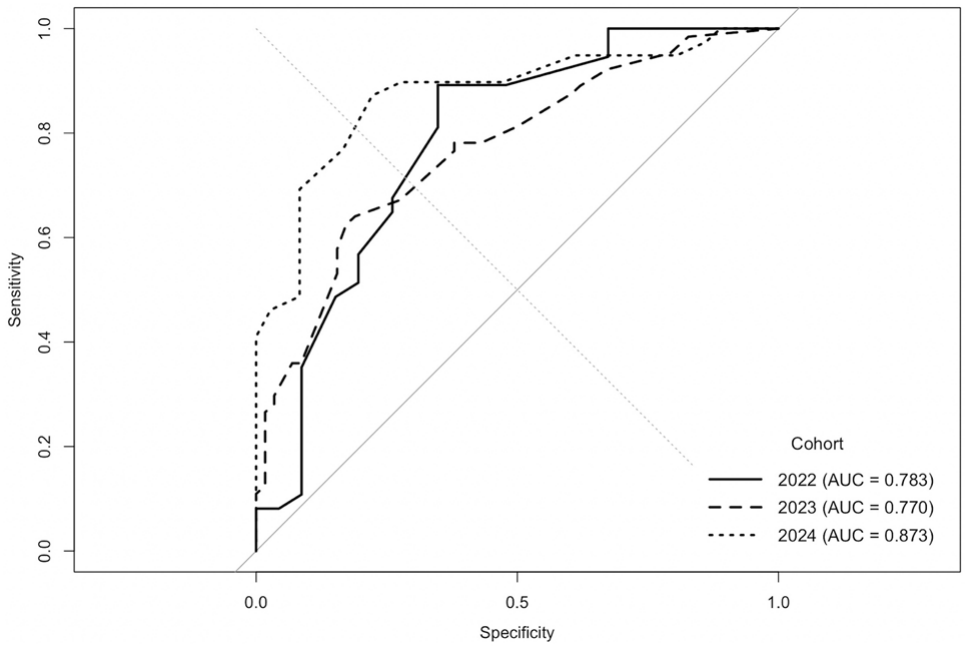
Temporal validation of nomogram performance. ROC curves demonstrating model discrimination across 2022, 2023, and 2024 cohorts. The model maintained stable performance with AUCs of 0.783 (2022), 0.770 (2023), and 0.873 (2024), confirming temporal robustness and generalizability

The model was developed in the 2023 cohort (*N* = 122) and temporally validated using 2022 (*N* = 83) and 2024 (*N* = 76) patients. Predictive discrimination remained strong across cohorts, with C-indices of 0.77 (95% CI 0.69–0.85), 0.78 (95% CI 0.68–0.88), and 0.87 (95% CI 0.79–0.96), respectively (Fig. 4). Pooled across all years (*N* = 281), overall C-index was 0.80 (95% CI 0.75–0.85), sensitivity 0.83, specificity 0.67, PPV 0.72, and NPV 0.80. Table 6 summarizes the nomogram performance across cohorts.

**Table 6:** Nomogram performance by development, temporal validation, and revisional cohorts

Cohort	N	LOS > 1 day	Threshold (Youden J)	Sens	Spec	PPV	NPV	C-index (AUC)	AUC 95% CI
Overall	281	0.50	0.50	0.83	0.67	0.72	0.80	0.804	0.753–0.855
2022 (temporal)	83	0.45	0.49	0.89	0.65	0.67	0.88	0.783	0.684–0.882
2023 (development)	122	0.53	0.56	0.63	0.83	0.80	0.67	0.770	0.687–0.852
2024 (temporal)	76	0.51	0.52	0.87	0.78	0.81	0.85	0.873	0.791–0.955
Primary only	234	0.49	0.50	0.81	0.68	0.70	0.79	0.789	0.731–0.847
Revisional/conversion only	47	0.57	0.56	0.93	0.65	0.78	0.88	0.876	0.780–0.972

*AUC* area under curve, *CI* confidence interval, *LOS* length of stay, *NPV* negative predictive value, *PPV* positive predictive value, *Sens* sensitivity, *Spec* specificity

### Model calibration

Calibration analysis showed excellent agreement between predicted and observed probabilities across all deciles of risk. The Hosmer–Lemeshow goodness-of-fit test supported model calibration (*χ*^2^ = 11.6, df = 8, *p* = 0.17). Overlaid calibration curves for 2022, 2023, and 2024 cohorts followed the ideal 45° line (Fig. S2), demonstrating temporal stability and consistent calibration.

### Sensitivity analysis for revisional surgery

A sensitivity analysis comparing primary vs. revisional/conversion procedures confirmed strong model performance in both settings. The model retained good discrimination in primary cases (AUC = 0.79) and excellent discrimination in revisional/conversion cases (AUC = 0.88), with notably high sensitivity (0.93) in the revisional/conversion subset. These findings support the model’s robustness across varying operative complexity and its generalizability to both standard and revisional/conversion bariatric populations (Fig. S3).

### Risk calculator

A web-based bedside calculator was developed using *R Shiny* application (https://michaeltolson.shinyapps.io/bariatric-delayed-discharge-2023/) to provide individualized predictions of delayed discharge risk using the five binary perioperative variables.

## Discussion

This study developed and temporally validated a simple, five-factor nomogram to predict delayed discharge after bariatric surgery using routinely available perioperative variables. The model demonstrated strong discrimination and calibration across three consecutive years, with AUC values ranging from 0.77 to 0.87, and retained excellent predictive performance in both primary and revisional/conversion procedures. At least within this single-center retrospective analysis, early postoperative tolerance, rather than patient-specific or preoperative factors, appears to drive discharge readiness after bariatric surgery. Consistent with this, BMI was the only preoperative factor independently associated with delayed discharge in multivariable modeling, yet it demonstrated poor discrimination on ROC analysis, underscoring that BMI alone is not a clinically useful predictor. Instead, physiologic and behavioral markers of postoperative readiness proved more informative. Two of the identified predictors, POD 1 hemoglobin decrease and reduced POD 0 oral intake, were anticipated by the authors based on observed institutional discharge patterns. It was surprising that preoperative factors did not have greater influence on the primary outcome, where baseline comorbidities would be expected to predict prolonged hospitalization, but perhaps this would be better investigated in a national database analysis with higher powered and diverse cohorts.

Although existing ERAS Society guidelines for perioperative care in bariatric surgery provide comprehensive recommendations spanning preoperative preparation through postoperative management, they do not provide recommendations for evaluating or determining discharge readiness [2]. The absence of defined objective parameters may contribute to variability in discharge practices across institutions. Our study adds to the literature by identifying select factors that may serve as actionable milestones. For example, oral intake demonstrated a quantifiable association with discharge timing, with a threshold of approximately 200 mL overnight emerging as a potential indicator of readiness. Incorporating such benchmarks into ERAS pathways can help set recovery goals for patients and lead to more standardized discharge decisions. Furthermore, early identification of patients who fail to meet these milestones can allow for targeted interventions, including optimization of antiemetic regimens, pain control, ambulation, and fluid management, thereby facilitating timely recovery and safe discharge.

Several prior studies have examined predictors of prolonged hospitalization after bariatric surgery, largely focusing on preoperative comorbidities and operative characteristics rather than perioperative recovery milestones. In one of the largest analyses to date, Fletcher et al. (2017) used National Surgical Quality Improvement Program data to evaluate more than 11,000 SG patients and identified advanced age, BMI > 50 kg/m^2^, anemia, chronic obstructive pulmonary disease, hypertension, renal insufficiency, and prolonged operative time as independent predictors of length of stay ≥ 3 days [8]. Similarly, Carter et al. [13] reported from a national gastric bypass cohort that diabetes, pulmonary disease, renal insufficiency, hypoalbuminemia, and longer operative times predicted hospitalization beyond 2 days [13]. Although these studies established key baseline risk factors, their reliance on administrative datasets limited the ability to evaluate granular postoperative parameters such as medication requirements, volume of oral intake, or laboratory changes that more directly reflect recovery readiness.

Institutional series have provided complementary insights into modifiable and process-related determinants of discharge. Meneveau et al. [9] demonstrated that case timing, surgeon experience, and postoperative testing practices significantly influenced length of stay, with routine upper GI swallow studies and afternoon operative starts independently associated with delayed discharge [9]. Major et al. [10] was the only other study identified in literature that included perioperative oral intake as a predictor for prolonged hospitalization, finding that every 100 mL of oral intake decreased the risk for delayed discharge by 23% [10]. Additionally, need for intravenous fluids and longer travel distance were predictors of prolonged hospitalization. Lastly, Nijland et al. [11] observed that depression, higher ASA class, sleeve gastrectomy, and postoperative complications independently prolonged stay in a cohort of over 1600 patients [11]. Collectively, these reports illustrate the multifactorial nature of prolonged hospitalization, reflecting variability in patient populations, institutional practices, and data resolution across studies. Despite these insights, standardized, quantitative markers of discharge readiness are insufficiently defined within ERAS pathways. Again, our study addresses this gap by introducing objective, early postoperative metrics that can be readily measured and incorporated into discharge decision-making.

This study possesses several strengths that enhance both methodological rigor and translational impact. First, it is one of few bariatric ERAS studies to include temporal validation, demonstrating the model’s reproducibility across consecutive patient cohorts. Temporal validation provides stronger evidence of generalizability than random internal validation, as it accounts for evolving case mix, surgeon experience, and workflow changes. Second, the model uses objective, binary predictors that can be measured early and consistently, reducing subjectivity and facilitating automation within electronic health records. Third, the study bridges data analytics with clinical application through the creation of an operational risk calculator. By providing instant risk estimates from routine postoperative inputs, this tool offers a scalable, user-friendly way to support discharge decisions in real time. Fourth, the analysis includes a revisional/conversion surgery sensitivity analysis, confirming that model performance remains strong even in higher-risk, technically complex cases. Finally, the study’s design emphasizes interpretability: each included variable is clinically meaningful, actionable, and consistent with established recovery milestones.

Several limitations warrant consideration. The study’s single-center, retrospective design may limit external generalizability, particularly to institutions with different ERAS protocols or resource constraints. Second, the model was validated temporally but not externally across institutions; multi-center validation would be the next step before widespread adoption. Third, while the five predictor variables were chosen for clinical simplicity, they may omit subtler contributors to delayed discharge, such as patient motivation, nursing workflow, or psychosocial readiness, which are all factors that are difficult to quantify retrospectively. Moreover, because our study was conducted within a military treatment facility, where most retirees and dependents bear less direct financial responsibility for hospitalization, some patients may have less incentive for earlier discharge, which could influence observed LOS patterns.

In conclusion, in this single-center, three-year retrospective analysis, a simplified five-variable nomogram accurately predicted delayed discharge following bariatric surgery, demonstrating robust discrimination and calibration across independent temporal cohorts. The model captures essential markers of early postoperative recovery, including operative efficiency, intravenous analgesic and antiemetic needs, oral intake tolerance, and hemoglobin stability, using data available within 24 h of surgery. Predictive accuracy was also stable across procedure types, including revisional and conversion cases. The resulting web-based risk calculator provides an actionable bedside tool to identify patients at risk for prolonged hospitalization, support early intervention, and enhance discharge planning within ERAS frameworks. Future multi-institutional, external validation and prospective integration into clinical decision support systems will be key steps toward broader implementation.

## Supplementary Information

Below is the link to the electronic supplementary material.

Supplementary file1 (JPG 198 KB)**Fig. S1** ROC curve for BMI as a sole predictor of delayed discharge. Receiver operating characteristic (ROC) analysis of body mass index (BMI) alone as a predictor of delayed discharge. BMI demonstrated minimal discriminatory ability (AUC=0.512) with an optimal Youden cutoff of 45 kg/m² (sensitivity 0.25, specificity 0.85), confirming BMI alone is a poor predictor relative to the multivariable model

Supplementary file2 (JPG 285 KB)**Fig. S2** Calibration by year: observed vs. predicted probability. Calibration plots by study year (2022–2024) showing observed vs. predicted risk of delayed discharge using LOESS-smoothed trends. The dashed diagonal line denotes perfect calibration. Across years, predicted probabilities closely tracked observed outcomes, demonstrating consistent calibration performance

Supplementary file3 (JPG 206 KB)**Fig. S3** Sensitivity analysis by procedure type. ROC curves comparing nomogram performance in all cases vs. primary and revisional/conversion bariatric surgery subsets. The model retained strong discrimination across groups with AUCs of 0.804 (all), 0.789 (primary), and 0.876 (revisional/conversion), underscoring model robustness in revisional/conversion surgery populations
