# Supplementary figures and images for: Development and temporal validation of a clinical nomogram to predict delayed discharge after bariatric surgery
Source: Surg Endosc. 2026 Mar 6;40(5):4158–68. doi: 10.1007/s00464-026-12663-2 (PMC13160949; doi:10.1007/s00464-026-12663-2)

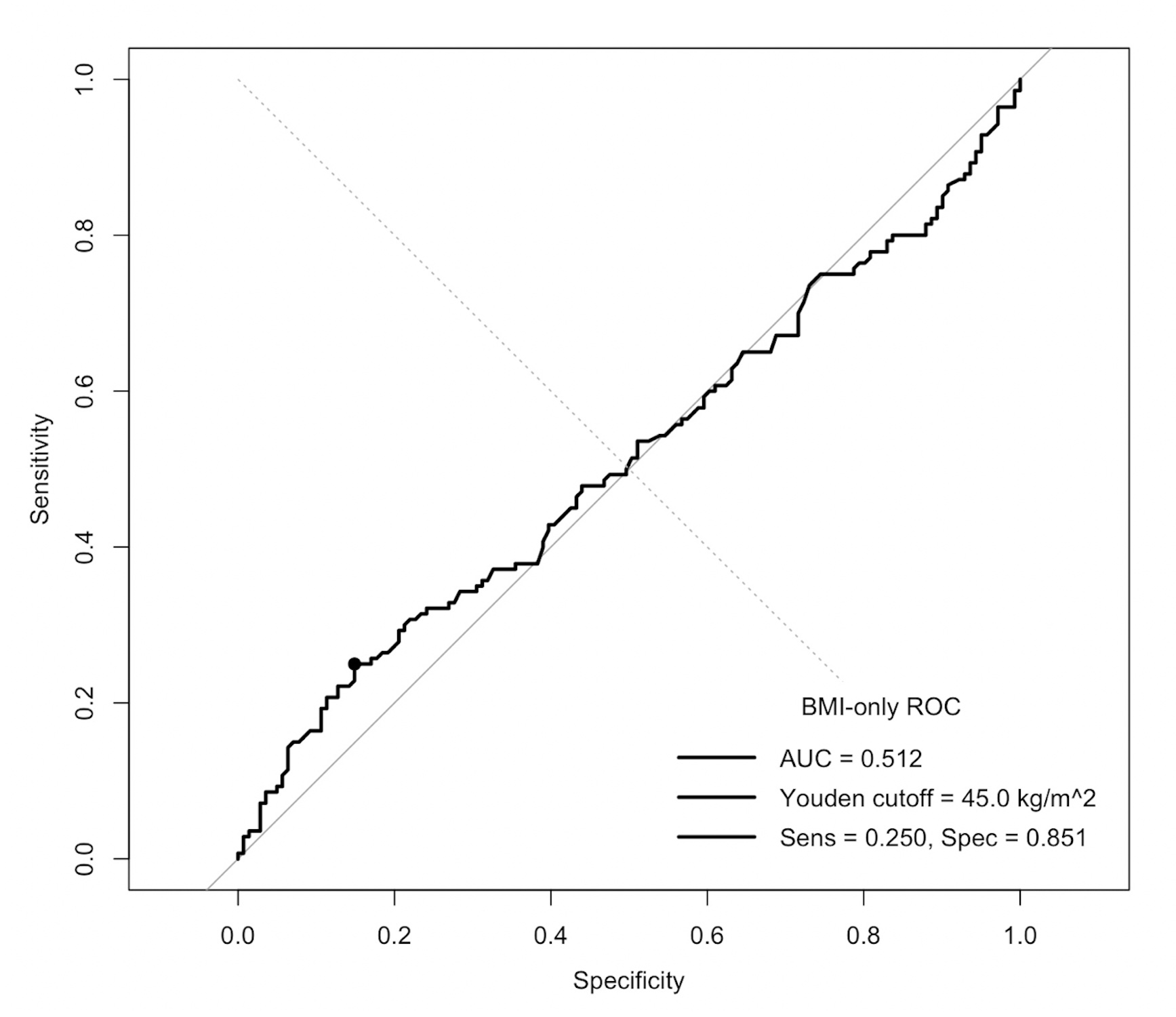

Supplement: Supplementary file 1 — Supplementary file1 (JPG 198 KB)Fig. S1 ROC curve for BMI as a sole predictor of delayed discharge. Receiver operating characteristic (ROC) analysis of body mass index (BMI) alone as a predictor of delayed discharge. BMI demonstrated minimal discriminatory ability (AUC=0.512) with an optimal Youden cutoff of 45 kg/m² (sensitivity 0.25, specificity 0.85), confirming BMI alone is a poor predictor relative to the multivariable model [file 464_2026_12663_MOESM1_ESM.jpg]

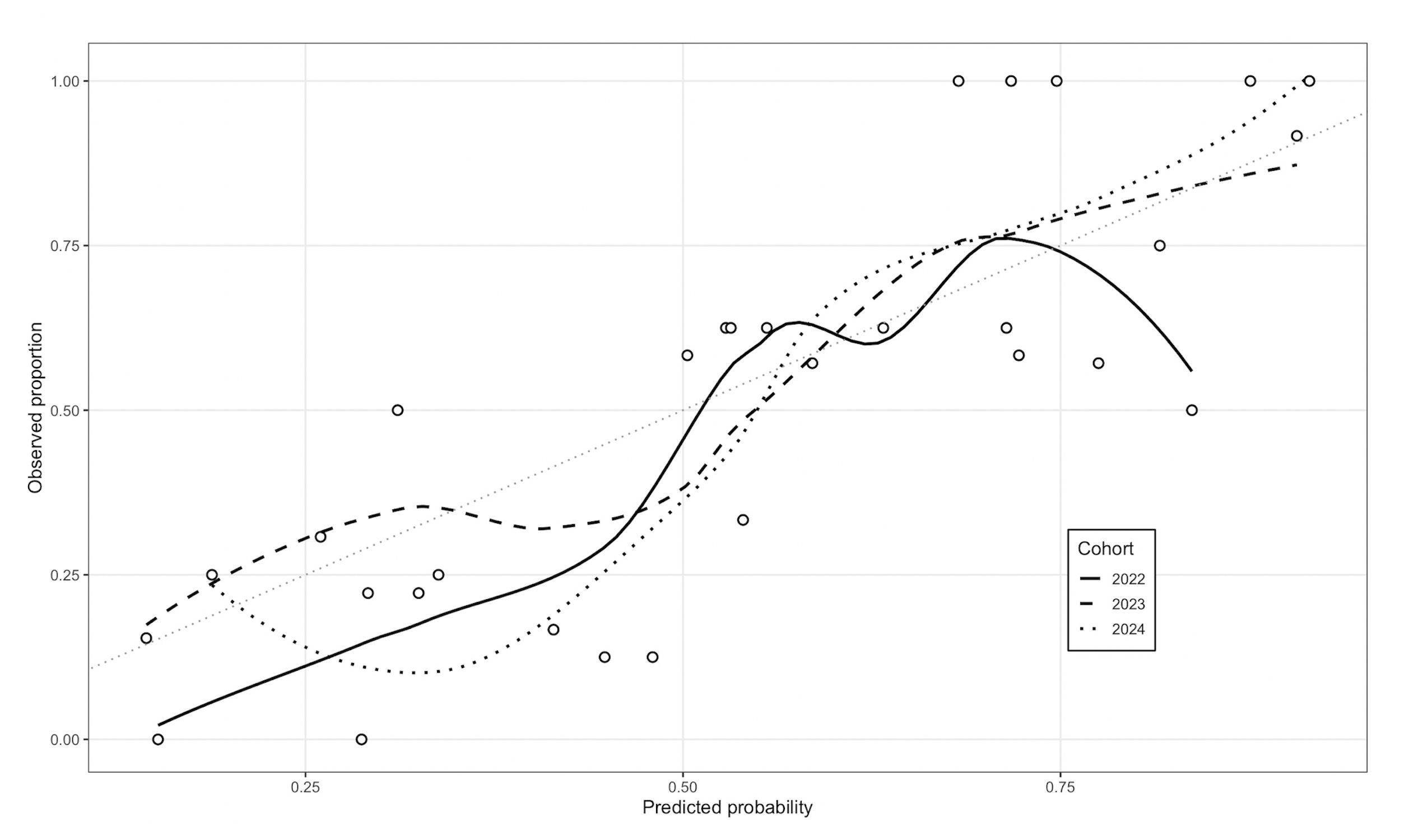

Supplement: Supplementary file 2 — Supplementary file2 (JPG 285 KB)Fig. S2 Calibration by year: observed vs. predicted probability. Calibration plots by study year (2022–2024) showing observed vs. predicted risk of delayed discharge using LOESS-smoothed trends. The dashed diagonal line denotes perfect calibration. Across years, predicted probabilities closely tracked observed outcomes, demonstrating consistent calibration performance [file 464_2026_12663_MOESM2_ESM.jpg]

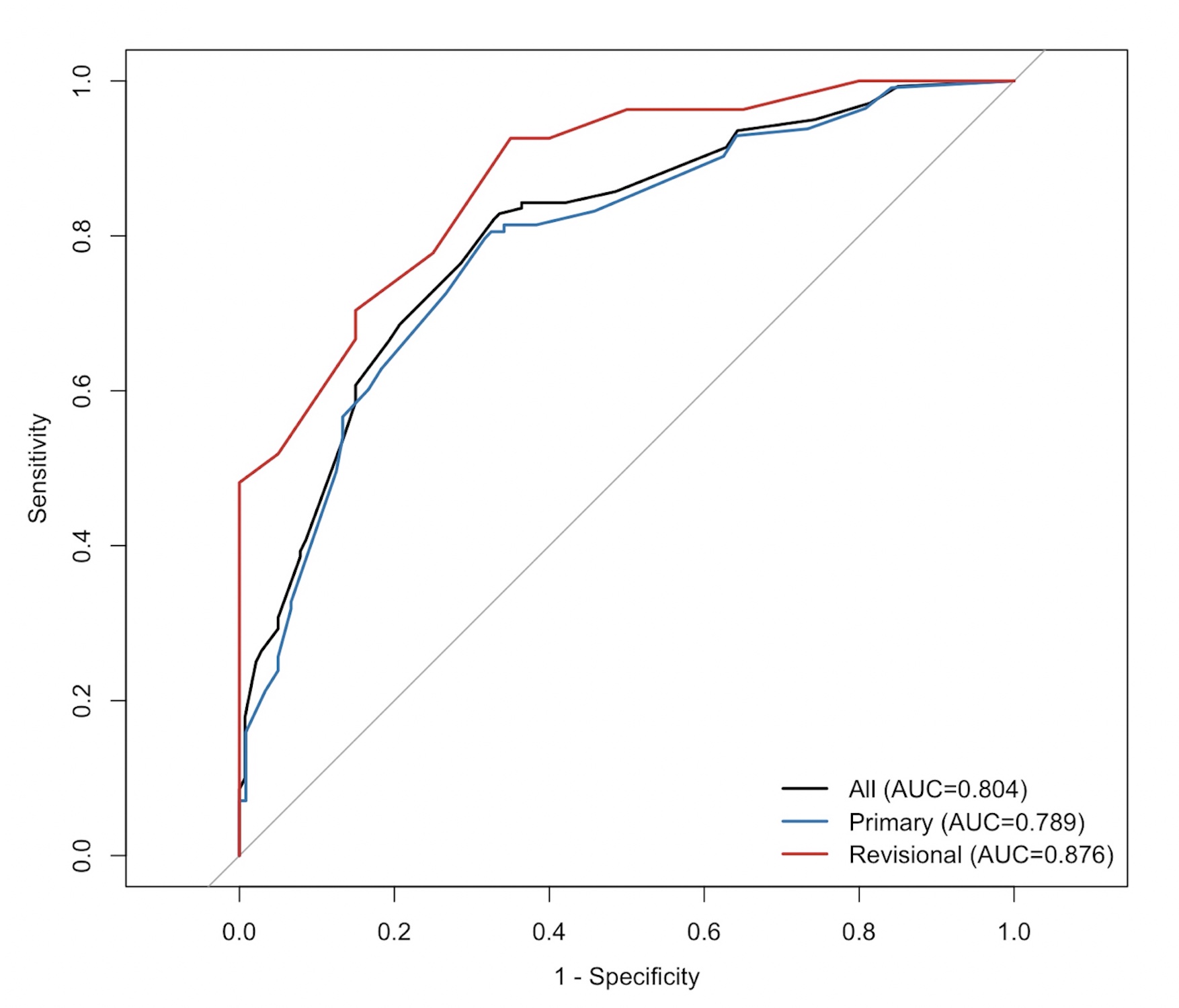

Supplement: Supplementary file 3 — Supplementary file3 (JPG 206 KB)Fig. S3 Sensitivity analysis by procedure type. ROC curves comparing nomogram performance in all cases vs. primary and revisional/conversion bariatric surgery subsets. The model retained strong discrimination across groups with AUCs of 0.804 (all), 0.789 (primary), and 0.876 (revisional/conversion), underscoring model robustness in revisional/conversion surgery populations [file 464_2026_12663_MOESM3_ESM.jpg]
